# Burnout among public health workers during the COVID-19 response: Results from a follow-up survey

**DOI:** 10.1371/journal.pmen.0000100

**Published:** 2024-08-07

**Authors:** Sarah Elizabeth Scales, Kristina W. Kintziger, Kahler W. Stone, Meredith A. Jagger, Jennifer A. Horney

**Affiliations:** 1 Department of Environmental, Agricultural & Occupational Health, University of Nebraska Medical Center, Omaha, NE, United States of America; 2 Department of Health and Human Performance, Middle Tennessee State University, Murfreesboro, TN, United States of America; 3 Independent Researcher, Austin, TX, United States of America; 4 Epidemiology Program, University of Delaware, Newark, DE, United States of America; University of Sousse Faculty of Medicine of Sousse: Universite de Sousse Faculte de Medecine de Sousse, TUNISIA

## Abstract

The public health workforce began the response to the COVID-19 pandemic with a critical workforce deficit, losing 20% of staff between 2018 and 2019. This study assesses changes in burnout among a cohort of 80 public health workers in the U.S. who completed multiple assessments during the ongoing COVID-19 response via Qualtrics. Self-reported burnout was assessed using a 5-point, validated, non-proprietary single-item measure. A binary burnout variable (1,2 = No; 3,4,5 = Yes) was created based on responses. Burnout direction (e.g., decreased, same, increased) was derived from changes in five-level work-related burnout scores. Factors indicating work-related exhaustion was higher among individuals reporting burnout in the follow-up survey compared to those without burnout. Baseline burnout, hours worked per week, and sleep quality were significant predictors of burnout at follow-up. A higher proportion of respondents reported burnout at follow-up than at the baseline survey. The ubiquitous nature of burnout among public health workers is a threat to the wellbeing of individuals in the workforce and the population’s health at large. Investments in expanding and supporting the public health workforce are needed.

## Introduction

The public health workforce started the response to the COVID-19 pandemic with a critical deficit, having lost 20% of its workforce between 2018–2019 [1]. To address the needs created by the pandemic, public health practitioners shouldered expanded responsibilities, took on more job roles, and worked more hours per day and days per week [2–5]. Despite reports from the de Beaumont Foundation that at least 80,000 additional public health workers were needed to ensure that state and local health departments could provide minimally acceptable levels of public health services to their respective communities, there are still critical staffing and funding shortages [6].

Burnout is defined by Maslach and Leiter (2016) as “a psychological syndrome emerging as a prolonged response to chronic interpersonal stressors on the job,” with three areas of effect, including 1) exhaustion, 2) cynicism and detachment, and 3) feelings of being ineffective or unaccomplished [7]. Lack of control over job circumstances, unclear expectations, challenging workplace dynamics, chaotic or extreme levels of work-related activity, lack of social support, and work-life imbalance also contribute to occupational burnout [8]. The COVID-19 pandemic response–in addition to the existing workforce and funding deficits–generated circumstances that exacerbated many of these factors, leading to high rates of burnout across many job sectors. While significant research has examined burnout among patient-facing healthcare providers such as nurses and physicians, the burnout experiences of the public health workforce have been underexplored [9,10].

Burnout and associated sequelae are a hindrance to the effectiveness of public health preparedness and response capacity. For example, in a U.S. public health workforce study conducted in September 2020, 66.2% of respondents reported burnout [5], and burnout was one of the most frequently cited reasons for considering leaving the public health workforce during the COVID-19 response [11]. Successful response to future public health emergencies is contingent upon a well-equipped and adequately supported public health workforce. Accordingly, we conducted a follow-up survey of public health workers in summer 2021 to assess changes in the prevalence of, and risk factors for, burnout among the U.S. public health workforce.

## Material and methods

### Data collection and cleaning

Data used in this study were collected in two cross-sectional surveys of U.S. public health practitioners–including epidemiologists, data scientists, program managers, and public health nurses, among others–predominantly from state and local public health departments. Respondents were recruited through professional networks and private social media channels. The baseline survey was open from August 12 through October 25, 2020, and the follow-up survey was open from June 16 through July 27, 2021. Surveys were distributed using Qualtrics (Provo, UT). Collected information covered demographics, personal well-being, career plans and trajectory, and work environment [5]. The survey is available as a supporting information (S1 File), and data are available in DesignSafe (PRJ-4545).

Participants who indicated willingness for follow-up were sent a second survey. Three hundred ninety participants responded to the first survey, 258 (66%) consented to follow-up and provided an email address, 85 (33%) respondents completed the follow-up survey, and 80 (31.0%) of those respondents completed all questions on burnout experiences. Data were cleaned by removing incomplete responses and linking baseline and follow-up surveys. For quality control, demographics were checked between surveys. Data from the baseline cross-sectional survey were retained for participants whose follow-up survey was rejected in the cleaning process if baseline survey data were complete.

### Ethics statement

The survey and all related materials were reviewed by the University of Delaware’s Institutional Review Board (IRB 1641836) and was determined to be exempt under Category 2 of the U.S. Department of Health and Human Services’ regulations for the protection of human subjects in research at 45CFR 46. As part of this review, a waiver of consent was approved and therefore, formal oral or written consent was not required. No medical records or archived samples were used.

### Burnout measure

A one-question, self-reported, 5-level burnout measure–developed and validated by Dolan et al., 2015, and psychometrically comparable to the validated, single-item Maslach Burnout Inventory Emotional Exhaustion (MBI:EE) subscale across clinical professionals–was used to assess burnout experience [12]. This measure is well-suited for measuring burnout and work-related exhaustion. These data were used to create a binary burnout variable (yes/no). Respondents who identified their experiences as ‘I enjoy my work’ or ‘occasionally I am under stress’ were categorized as not experiencing burnout. Those who responded as ‘I am definitely burning out,’ ‘I have the symptoms of burnout,’ and ‘I feel completely burned out’ were categorized as experiencing burnout.

### Models

Data were downloaded from Qualtrics and cleaned and analyzed in SAS Studio (Cary, NC). Chi-square and Fisher’s exact tests at the 0.05 level of significance were used to compare the distribution of risk factors between the baseline and follow-up survey. McNemar’s test was used to compare burnout experience for respondents to both surveys. Bivariate logistic regression was used to assess the strength of association between binary work and exhaustion-related risk factors for burnout assessed only at follow-up Odds ratios (OR) and 95% confidence intervals (CIs) are reported.

Multivariable generalized linear regression was used to describe the effects of baseline burnout (yes/no), years in the public health workforce (5+ years/ <5 years), hours worked per week (<40 hours/ 40–49 hours/ 50+ hours per week), quality of sleep (never or rarely well/ frequently or occasionally well) on burnout at follow-up. The model was specified using PROC GENMOD in SAS Studio (Cary, NC) with a binary distribution, logit link, and compound symmetry, and accounted for repeated measures of burnout at baseline and follow-up. Adjusted odds ratios (AOR) and 95% CIs are reported.

## Results

### Demographic and work-related characteristics comparing baseline and follow-up cohorts

For quality control, demographics were checked for consistency between baseline and follow-up surveys. Basic demographics, well-being, and work-related factors were comparable between the baseline and follow-up surveys. Descriptive statistics and comparisons for baseline and follow-up cohorts are summarized in **Table 1.**

**Table 1:** Demographic characteristics of respondents in the initial survey and follow-up survey, including χ2 p-values comparing proportions of given demographic characteristics for respective survey waves.

		Initial Survey Only		Follow-Up		
		N	%	N	%	χ2 p-value
**Age at Survey **						
** **	18–29	62	20.33	16	18.82	0.95
** **	30–39	109	35.74	32	37.65	
** **	40–49	71	23.28	18	21.18	
** **	50+	63	20.66	19	22.35	
**Gender **						
** **	Female	245	80.33	69	81.18	0.83*
** **	Male	52	17.05	13	15.29	
** **	Other/Missing	8	2.62	3	3.53	
**Race **						
** **	Other	65	21.31	13	15.29	0.22
** **	White	240	78.69	72	84.71	
**Years in Public Health **						
** **	< 5 years	100	32.79	29	34.12	0.83
** **	5+ years	204	66.89	56	65.88	
**Education **						
** **	Bachelor	36	11.8	9	10.59	0.64*
** **	Doctorate	65	21.31	16	18.82	
** **	Master	172	56.39	52	61.18	
** **	Other/Missing	17	5.57	2	2.35	
** **	Professional	15	4.92	6	7.06	
**Marital Status **						
** **	Married/Partnered	185	60.66	50	58.82	0.48
** **	Not Partnered	120	39.34	35	41.18	
**Region **						
** **	Midwest	18	5.9	4	4.71	0.83
** **	Northeast	27	8.85	6	7.06	
** **	South	87	28.52	23	27.06	
** **	West	168	55.08	52	61.18	
**Ethnicity**	Hispanic	23	7.54	11	12.94	0.12
** **	Non-Hispanic, Prefer not to answer, Missing	282	92.46	74	87.06	

*Fisher’s Exact reported.

### Follow-up cohort

The follow-up cohort was demographically different than the public health workforce (e.g., more female, Whiter, and less Hispanic/Latinx-identifying). Females were overrepresented among the respondents to the follow-up survey (81% versus 79% of public health workforce). Follow-up respondents were more likely to be White (75% versus 54%) and less likely to be identify as Hispanic or Latinx (13% versus 18%) [13]. Survey respondents were younger overall, with most respondents between 30 and 39 years of age, compared to a mean age of 46 years for the workforce [14]. The majority of follow-up respondents had master’s degrees (80%) and job roles in communicable disease areas including COVID-19 (57%).

### Experience of burnout among follow-up cohort

The proportion of respondents with burnout in the baseline survey (60.4%) was significantly lower than the proportion reporting burnout at both the baseline and follow-up survey (χ2 = 4.37; p-value = 0.0367). Individuals reporting burnout in the baseline survey were more than 7 times as likely to report burnout at follow-up when compared to those reporting no baseline burnout (OR = 7.26; 95% CI: 2.43–21.71; McNemar’s χ2 = 4.54; p-value = 0.03). Over half of respondents to both surveys reported burnout in both; 20% reported burnout at only baseline survey; 7.5% reported burnout at follow-up but not baseline survey; and 21.25% reported did not report burnout in either survey.

Follow-up survey respondents reported feeling emotionally exhausted from work, frustrated with work, and burnout. Nearly all (97.87%) of individuals reporting burnout reported being exhausted at the end of the day; they also noted feeling exhausted every hour of the day (72.34%) and in the morning (87.23%). Those with and without burnout significantly differed in the experience of tiredness every hour of the day (OR: 6.02; 95% CI: 2.26–16.02) and exhaustion in the morning (OR: 10.51; 95% CI: 3.48–31.75). Nearly 90% of individuals without burnout reported having enough energy for family and friends during leisure time compared to only 70% of those reporting burnout (OR: 0.33; 95% CI: 0.10–1.10). Follow-up survey respondents were 76% more likely to report burnout at the baseline survey than those only participating in the baseline survey (OR = 1.76; 95% CI: 1.03–3.01). Findings are summarized in **Table 2.**

**Table 2:** Burnout-related variables of interest for the follow-up cohort of public health workers.

Risk Factor		Burned Out		Not Burned Out		OR (95% CI)
		N	%	N	%	
**Work is emotionally exhausting **	Yes	39	82.98	16	48.48	5.18 (1.86–14.39)
	No	8	17.02	17	51.52	
**Work causes burnout **	Yes	43	91.49	11	33.33	21.50 (6.13–75.37)
	No	4	8.51	22	66.67	
**Work is frustrating **	Yes	35	74.47	15	45.45	3.50 (1.36–9.04)
	No	12	25.53	18	54.55	
**Feeling worn out at the end of the working day **	Yes	46	97.87	27	81.82	8.52 (0.94–76.80)
	No	1	2.13	5	15.15	
**Exhausted in the morning at the thought of another day at work **	Yes	41	87.23	13	39.39	10.51 (3.48–31.75)
	No	6	12.77	20	60.61	
**Feeling every hour that work is tiring.**	Yes	34	72.34	10	30.3	6.02 (2.26–16.02)
	No	13	27.66	23	69.7	
**Enough energy for family and friends during leisure time.**	Yes	33	70.21	29	87.88	0.33 (0.10–1.10)
	No	14	29.79	4	12.12	

A summary of the multivariable logistic regression model is found in **Table 3**. Participants with five or more years of experience in the public health workforce were less likely to report burnout at follow-up (AOR = 0.45; 95% CI: 0.13–1.53) when controlling for baseline burnout, hours worked per week, and sleep quality. Those reporting frequently or occasionally getting enough sleep vs. rarely or never getting enough sleep had lower adjusted odds of burnout (AOR: 0.12, 95% CI: 0.03–0.52) when adjusting for baseline burnout, experience, and hours worked per week. When adjusting for baseline burnout, experience, and sleep quality, there was no difference in burnout at follow-up for respondents working less than 40 hours and 41–49 hours per week (AOR = 0.96; 95% CI: 0.16–5.82); those working 50+ hours per week were three times as likely to report burnout than colleagues working 40 or less hours per week (AOR = 3.02; 95% CI: 0.80–11.48). Baseline burnout was predictive of burnout at follow-up, where individuals without baseline burnout were more likely to continue to report no burnout at follow-up compared to those with baseline burnout when adjusting for experience, sleep quality, and hours worked per week (AOR: 7.76; 95% CI: 2.03–29.65).

**Table 3:** Summary of point estimates burnout at the follow-up survey from the multivariable generalized linear regression assessing the impact of wellbeing, workplace, and demographic variables on burnout at follow-up. As noted above, odds ratios and 95% confidence intervals represent the odds of burnout described when controlling for other variables included in the model.

Effect	OR	95% CI
Initial Burnout vs. No Initial Burnout	7.76	2.03–29.65
5+ Years of Public Health Work vs. <5 Years of Public Health Work	0.45	0.13–1.53
41–49 hours/week vs. <40 hours/week	0.96	0.16–5.82
50+ hours/week vs. <40 hours/week	3.02	0.80–11.48
Frequently or Occasionally Sleep Well vs. Rarely or Never Sleep Well	0.12	0.03–0.52

## Discussion

There is limited literature on burnout among the public health workforce–predominantly composed of non-patient-facing practitioners–specifically. A 2024 meta-analysis of studies from the U.S., South Korea, Japan, Malaysia, China, and India, found that nearly 40% of public health practitioners were experiencing burnout [15]. In our study, the prevalence of burnout was high in both baseline (165 of 273) and follow-up surveys (47 of 80), with roughly 60% of respondents reporting burnout. Individuals with burnout at follow-up reported experiencing symptoms in-line with the Maslach and Leiter’s “three key dimensions” of burnout–exhaustion, detachment, and ineffectiveness [7]. For example, a higher proportion of individuals who reported burnout at follow-up indicated that work was emotionally exhausting, causes burnout, and causes frustration, compared to those not reporting burnout. Work was more tiring, and workdays were more exhausting at both the beginning and end of the day for those reporting burnout. Further, individuals experiencing burnout had less energy for their family, friends, and hobbies. These findings are consistent with other cross-sectional studies that reported high prevalences of burnout among healthcare workers. For example, 67% of healthcare workers in a multinational study conducted between March and June of 2020 reported burnout [16]. Other longitudinal studies examining the trajectory of burnout later in the course of the COVID-19 pandemic also noted that healthcare workers continued to report high levels of psychological distress and mental health impacts as the pandemic progressed [17,18]. The persistence of burnout was seen among respondents in our study.

Experience, job roles, and hours worked can be important risk factors for burnout. Hours worked could be an important indicator of ability to maintain work-life balance, which has been identified as a key risk factor for burnout during the COVID-19 response [2,19]. In a study of Intensive Care Unit staff, burnout increased nearly 80% for physicians and nurses between late 2019 and June 2020, with the highest increase among those working the most overtime and those caring for patients with COVID [20]. Working more than 60 hours per week has consistently been shown to lead to workplace burnout [21]. Our study showed that odds of burnout were lower for respondents working less than 50 hours a week, although there was not an appreciable difference in burnout experience for those working less than 40 and those working 40–49 hours per week. Emerging literature highlights the potential value of flexible scheduling for mitigating burnout [22]. Other studies, conducted predominantly in European populations, have shown both positive and negative aspects of remote and hybrid work environment for addressing burnout [23,24]. Acceptable overtime and capped work hours can help health departments to maintain productivity while also protecting their workforce against burnout and additional stressors. Both flexible scheduling and hybrid/remote work options should be further explored as approaches for controlling work hours, giving employees occupational autonomy, and effectively reducing burnout.

Studies have found burnout to be highest in trainees and residents and to decrease with age and experience [25,26], with some bimodal findings for age and burnout [27]. In our study, respondents with five or more years of public health work, while not statistically significant, were less likely to report burnout. This could be attributable to a range of factors. For example, public health practitioners took on a range of additional job roles throughout the pandemic [5,28]. Individuals with more years of experience could have skills developed through prior experiences and utilize more adept professional coping strategies compared to those with less time and experience in the workforce. Mentoring, which is often a function of longevity in the workforce, has been shown to improve experience of burnout for both mentors and mentees [29]. For mentors in-specific, this relationship could improve feelings of burnout such as limited effectiveness and detachment. Tailored supportive measures across career stages should be explored as preemptive steps for reducing risk of developing and severity of burnout.

Prior to the COVID-19 pandemic, a small-scale survey found that insufficient sleep was a predictor of burnout [30]. Sleep can also mitigate the negative mental health impacts of stressful situations and work environments, while a lack of sleep can augment feelings of burnout [31]. This was reflected among our study respondents. However, without additional information about individual sleep habits and changes in sleep habits due to the pandemic, it is difficult to discern the role sleep played in feelings of burnout among our cohort. Future research should more closely address the relationship between subjectively or objectively measured sleep quality and burnout.

Our choice of burnout measure could have implications for study findings. The non-proprietary burnout measure we chose for our study has been validated across a number of occupations within the health sector and compared against the MBI:EE [12]. The MBI:EE is a subscale within the 22-item Maslach Burnout Inventory (MBI)–the gold standard of burnout measures [32]. There are several considerations for why we chose to use the non-proprietary measure rather the MBI or MBI:EE. First, the measure used in our study is non-proprietary while the MBI is proprietary. Second, and most importantly, a 22-item scale was less feasible for self-reported surveys. Given that the non-proprietary single question, 5-item measure has been validated for use in health-related professions and has a sensitivity of 83.2% and specificity of 87.4%, we felt that this measure’s performance and ease-of-use made it the best fit for our methods, study population, and research questions. There could be measurement errors and resulting biases stemming from our choice of measure.

There are several important limitations to this study. Our study has a relatively small sample size, in part due to needs and challenges specific to the different phases of pandemic response at the time of both baseline and follow-up survey deployment. Small cell counts made more granular analysis challenging. The follow-up cohort was appreciably smaller than the baseline cohort, with a response rate of only 33%. This concern was mitigated by no significant differences in the distribution of key demographic variables. However, selection bias could have influenced our findings if those most affected by COVID-19-associated burnout were more likely to respond to the follow-up survey (see *Experience of burnout among follow-up cohort)*. Conversely, response rates could vary by work-related stressors, with those who were less affected by these stressors having more time to complete the survey than those experiencing higher levels of burnout. Our survey respondents overrepresented female, White, and younger demographics compared to the overall public health workforce. While the public health workforce is predominantly White and female [13], the overrepresentation of these demographics in our data render our findings not generalizable to the workforce at large. Additionally, the mechanisms that underpin burnout and individual experiences of burnout are complicated and multimodal. Our study accounts for some, but not all, of the potential confounders that could be influence the development of burnout among respondents.

Future studies should better represent the workforce at large. This will require a better census of the public health workforce, including contact information being made accessible through professional bodies such the National Association of County and City Health Officials (NACCHO). Qualitative research, building on our team’s prior work using key informant interviews, could add context to findings in this study [3,4]. Despite these limitations, this study adds value to the existing literature and gives context to the discussion of burnout experiences of the public health workforce throughout the COVID-19 pandemic.

## Conclusions

Broadly, the burnout experiences of the public health workforce have been underexplored. Our study reported high levels of burnout among public health practitioners at multiple time points throughout the COVID-19 pandemic. Individuals experiencing burnout were, as expected, more likely to report experiencing work-related exhaustion. Participants with five or more years of experience and working less than 50 hours per week were less likely to report burnout. Findings regarding sleep quality and burnout were inverse from the expected relationship and garner further investigation. Workforce mental health and wellbeing must be considered to address workforce deficits and bolster public health preparedness and response capacity for future emergency response. Further investment in occupational research among public health practitioners is needed to identify and implement evidence-informed policies to address burnout within the workforce.

## Supporting information

S1 File Survey distributed to public health practitioners, June 16 through July 27, 2021. (PDF)
