# Supplementary material for: Burnout among public health workers during the COVID-19 response: Results from a follow-up survey
Source: PLOS Ment Health. 2024 Aug 7;1(3):e0000100. doi: 10.1371/journal.pmen.0000100 (PMC12798593; doi:10.1371/journal.pmen.0000100)
Supplement: S1 File — (PDF) [file pmen.0000100.s001.pdf]

PH Practitioner COVID-19 Survey

Start of Block: Consent

Q1
The public health response to the SARS-CoV-2 (COVID-19) pandemic is unprecedented in recent history; as such, it is taking a toll on the health and well-being of essential workers. As the response will likely continue for months and intensify in some areas, understanding the on-going impacts on public health practitioners will allow us to modify and adapt policies and procedures to allow for a more sustained and healthy workforce.

The goal of this longitudinal survey is to systematically assess the impacts of the COVID-19 response on the mental and physical health of epidemiologists and other public health workers, identify risk and protective factors for burnout within workplaces and communities, understand short- and long-term career decisions that may result from the pandemic and response, among other areas of interest.

The target population of this longitudinal assessment includes epidemiologists and those working in public health performing epidemiology-related functions who have been directly and indirectly impacted by the public health response to COVID-19. This survey was reviewed by the University of Delaware and determined to be exempt (IRB#1641836-1) .

You are being contacted because you participated in the first survey and indicated that you were interested in participating in a follow-up survey.

Q2 Do you wish to participate in this follow-up assessment?

○ Yes (1)

○ No (2)

Skip To: End of Survey If Do you wish to participate in this follow-up assessment? = No

---

Q3 Please verify your name and contact information.

- ○ Name (1) ____
- ○ Email (2) ____
- ○ Organization or Agency (3) ____
- ○ Title/Position (4) ____
- ○ Program or Department (5) ____

End of Block: Consent

Start of Block: Background Information

Q4 What is your age?

- ○ 18-29 (1)
- ○ 30-39 (2)
- ○ 40-49 (3)
- ○ 50-64 (4)
- ○ 65-74 (5)
- ○ 75-84 (6)
- ○ 85+ (7)
- ○ Prefer not to say (8)

Q5 What is your gender?

- ○ Male (1)
- ○ Female (2)
- ○ Other (Please Specify) (3) ____
- ○ Prefer not to say (4)

Q6 Are you of Spanish or Latino origin?

- ○ Yes (1)
- ○ No (2)
- ○ Prefer not to say (3)

Q7 What would best describe your race?

- ○ American Indian/Alaskan Native (1)
- ○ Asian (2)
- ○ Black (3)
- ○ Native Hawaiian/Other Pacific Islander (4)
- ○ White (5)
- ○ Other (please specify) (6) ____
- ○ Prefer not to say (7)

Q8 What is your martial status?

○ Currently Married/Partnered (1)

○ Widowed (2)

○ Divorced (3)

○ Separated (4)

○ Never Married (5)

○ Other (please specify) (6) ____

○ Prefer not to say (7)

Q9 How many people currently reside in your household including yourself?

○ Adults (18 years and older; including yourself) (1)
____

○ Children (less than 18 years old) (2)
____

○ Prefer not to say (3) ____

Q10 What is your state or territory of residence?

▼ Alabama (1) ... U.S. Virgin Islands (55)

End of Block: Background Information

Start of Block: Public Health Role and Training

Q12 Is your position Full Time or Part Time?

▼ Full Time (1) ... Prefer not to say (3)

Q13 Are you a new hire in the public health field since March 2020?

- ◯ Yes (1)
- ◯ No (2)

Q14 What public health setting do you currently work in?

- ◯ Public Health Practice (1)
- ◯ Academic (2)
- ◯ Clinical Setting (3)
- ◯ Private/Industry (4)
- ◯ Non-Profit Organization (5)
- ◯ Other: (6) ____

Skip To: Q60 If What public health setting do you currently work in? != Public Health Practice

---

Q15 If Public Health Practice, by what level of government are you employed? (For example, if you are a CDC assignee to a local public health department, please select Federal).

- ◯ Federal (1)
- ◯ State (2)
- ◯ Territorial (3)
- ◯ Local/County/Regional (4)
- ◯ Other: (5) ____

Q61 If Public Health Practice, what level of community do you serve? (For example, if you are a CDC assignee to a local public health department, please select Local/County/Regional).

- Federal (1)
- State (2)
- Territorial (3)
- Local/County/Regional (4)
- Other (please specify) (5) ____

Q60 Are you a contract employee (e.g., CSTE trainee, contracted from a school of public health or private agency to work at or for a local or state health department)?

- Yes (1)
- No (2)
- Other (please specify) (3) ____

Q16 How many years of experience do you have in public health?

- less than 1 year (1)
- 1-4 years (2)
- 5-9 years (3)
- 10-14 years (4)
- 15+ years (5)

Display This Question:

If Are you a new hire in the public health field since March 2020? = No

Q17 What was your pre-pandemic **program area**? (Select all that apply)

- ▢ Chronic Disease (1)
- ▢ Environmental Health (2)
- ▢ Genomics (3)
- ▢ Infectious Disease (4)
- ▢ Informatics (5)
- ▢ Injury (6)
- ▢ Maternal and Child Health (7)
- ▢ Mental Health (8)
- ▢ Occupational Health (9)
- ▢ Preparedness (10)
- ▢ Substance Use Disorders (11)
- ▢ Vital Statistics (12)
- ▢ Sexually Transmitted Infections/Sexual Health (13)
- ▢ Tuberculosis (14)
- ▢ Other (Please Specify) (15) ____
- ▢ None, I did not work in any program area (16)

Q18 What was your pre-pandemic **public health role**? (select all that apply)

- ▢ Administration (1)
- ▢ Benefits Coordinator (2)
- ▢ Disease Investigator (3)
- ▢ Evaluation (4)
- ▢ Health Educator (5)
- ▢ Planning/Preparedness (6)
- ▢ Policy (7)
- ▢ Program Manager/Project Coordinator (8)
- ▢ Surveillance (9)
- ▢ Research (10)
- ▢ Other (please specify) (11) ____
- ▢ None, I did not work in any public health role (12)

Q19 What is your <u>current</u> **program area**? (select all that apply)

- ☐ Chronic Disease (1)
- ☐ COVID-19 Specific (2)
- ☐ Environmental Health (3)
- ☐ Genomics (4)
- ☐ Infectious Disease (5)
- ☐ Informatics (6)
- ☐ Injury (7)
- ☐ Maternal and Child Health (8)
- ☐ Occupational Health (9)
- ☐ Preparedness (10)
- ☐ Substance Use Disorders (11)
- ☐ Vital Statistics (12)
- ☐ Sexually Transmitted Infections/Sexual Health (13)
- ☐ Tuberculosis (14)
- ☐ Other (please specify) (15) ____
- ☐ None, I do not work in any program area (16)

Q20 What is your <u>current</u> **public health role**? (select all that apply)

- ☐ Administration (1)
- ☐ Benefits Coordinator (2)
- ☐ COVID-19 Specific (3)
- ☐ Disease Investigator (4)
- ☐ Evaluation (5)
- ☐ Health Educator (6)
- ☐ Planning/Preparedness (7)
- ☐ Policy (8)
- ☐ Program Manager/Program Coordinator (9)
- ☐ Surveillance (10)
- ☐ Research (11)
- ☐ Other (please specify) (12) ____
- ☐ None, I do not work in any public health role (13)

Page Break

Q62 How have your current work-related responsibilities changed compared to your pre-pandemic work-related responsibilities?

	Decrease (1)	No Change (2)	Increase (3)
Program Area (1)	○	○	○
Role (2)	○	○	○
Amount of Work (in hours) (3)	○	○	○

Q22 What is the highest degree or level of education you have completed?

▢ Less than high school diploma (1)

▢ High school degree or equivalent (2)

▢ Technical certificate/training (please specify): (3) ____

▢ Associate's degree (e.g., AA, AS) (4)

▢ Bachelor's degree (e.g., BA, BS, BSN; please specify subject): (5) ____

▢ Masters degree (e.g., MPH, MSPH, MS, MBA, NP, MSN; please specify degree and subject): (6) ____

▢ Professional degree beyond a bachelor's degree (e.g., MD, DDS, DVM, DNP; please specify degree and specialty, if applicable): (7) ____

▢ Doctorate degree (e.g. PhD, DrPH; please specify degree and subject): (8) ____

Q23 Do you have any other certifications or training related to your role in public health?

- [ ] CHES (1)
- [ ] CPH (2)
- [ ] GISP (3)
- [ ] CIC (4)
- [ ] Other (please specify) (5) ____

Q24 In January 2020, which describes your career plans or trajectory with regards to public health?

- ○ <u>Leave</u> or <u>retire</u> **during 2020** (1)
- ○ <u>Remain</u> **during 2020**, <u>but leave or retire</u> in a **year or two** (2)
- ○ <u>Remain</u> for **3 or more years** (3)
- ○ Undecided (4)
- ○ Not applicable (5)
- ○ Prefer not to say (6)

Q25 As of today, what are your career plans or trajectory with regards to public health?

- Leave or retire **during 2021** (1)
- Remain **during 2021**, but leave or retire in a **year or two** (2)
- Remain for **3 or more years** (3)
- Undecided (4)
- Not applicable (5)
- Prefer not to say (6)

End of Block: Public Health Role and Training

Start of Block: Personal Health and Well-Being

Q26 Thinking about your physical health, which includes physical illness and injury, for how many days during the past 30 days was your physical health not good?

- Number of days: (1) ____
- None (2)
- Don't know/ not sure (3)
- Prefer not to say (4)

Q27 Thinking about your mental health, which includes stress, depression, and problems with emotions, for how many days during the past 30 days was your mental health not good?

- Number of days: (1) ____
- None (2)
- Don't know/ not sure (3)
- Prefer not to say (4)

Q28 During the past 30 days, for about how many days did poor physical or mental health keep you from doing your usual activities, such as self-care, work, or recreation?

- ○ Number of days: (1) ____
- ○ None (2)
- ○ Don't know/ not sure (3)
- ○ Prefer not to say (4)

Page Break

Q64 Over the last 30 days, how often have you been bothered by any of the following problems?

	Not bothered at all (1)	Bothered a little (2)	Bothered a lot (3)
Stomach pain (1)	○	○	○
Back pain (2)	○	○	○
Pain in you arms, legs, or joints (knees, hips, etc.) (3)	○	○	○
Menstrual cramps or other problems with your periods, if applicable (4)	○	○	○
Headaches (5)	○	○	○
Chest pain (6)	○	○	○
Dizziness (7)	○	○	○
Fainting spells (8)	○	○	○
Feeling your heart pound or race (9)	○	○	○
Shortness of breath (10)	○	○	○
Pain or problems during sexual intercourse (11)	○	○	○
Constipation, loose bowels, or diarrhea (12)	○	○	○
Nausea, gas, or indigestion (13)	○	○	○
Feeling tired or having low energy (14)	○	○	○

Trouble sleeping (15)	○	○	○

Page Break

Q65 Please rate the current (i.e., last two weeks) severity of sleep problem(s) or insomnia.

	None (1)	Mild (2)	Moderate (3)	Severe (4)	Very severe (5)
Difficulty falling asleep (1)	○	○	○	○	○
Difficulty staying asleep (2)	○	○	○	○	○
Problems waking up too early (3)	○	○	○	○	○

Q66 How satisfied/dissatisfied are you with your current sleep pattern?

- ○ Very Satisfied (1)
- ○ Satisfied (2)
- ○ Moderately Satisfied (3)
- ○ Dissatisfied (4)
- ○ Very Dissatisfied (5)

Q67 How noticeable to others do you think your sleep problem is in terms of impairing the quality of your life?

- Not at all noticeable (1)
- A little (2)
- Somewhat (3)
- Much (4)
- Very much noticeable (5)
- Not applicable (6)

Q68 How worried/distressed are you about your current sleep problem?

- Not at all worried (1)
- A little (2)
- Somewhat (3)
- Much (4)
- Very much worried (5)
- Not applicable (6)

Q69 To what extent do you consider your sleep problem to interfere with your daily functioning (e.g., daytime fatigue, mood, ability, to function at work/daily chores, concentration, memory, mood, etc.) currently?

- Not at all interfering (1)
- A little (2)
- Somewhat (3)
- Much (4)
- Very much interfering (5)
- Not applicable (6)

Q29 Over the last 2 weeks, how often have you been bothered by the following problems?

	Not at all (1)	Several days (2)	Over half the days (3)	Nearly every day (4)
Feeling nervous, anxious, or on edge (1)	○	○	○	○
Not being able to stop or control worrying (2)	○	○	○	○
Worrying too much about different things (3)	○	○	○	○
Trouble relaxing (4)	○	○	○	○
Being so restless that its hard to sit still (5)	○	○	○	○
Becoming easily annoyed or irritable (6)	○	○	○	○
Feeling afraid as if something awful might happen (7)	○	○	○	○
Little interest or pleasure in doing things (8)	○	○	○	○
Feeling down, depressed, or hopeless (9)	○	○	○	○

Page Break

Q30 Overall, based on your definition of burnout, how would you rate our level of burnout today?

- I enjoy my work. I have no symptoms of burnout. (1)
- Occasionally I am under stress, and I don't always have as much energy as I once did, but I do not feel burned out. (2)
- I am definitely burning out and have one or more symptoms of burnout, such as physical and emotional exhaustion. (3)
- The symptoms of burnout that I'm experiencing wont go away. I think about frustration at work a lot. (4)
- I feel completely burned out and often wonder if I can go on. I am at the point where I may need some changes or may need to seek some sort of help. (5)

Q70 Please rate your work-related burnout.

	To a very high degree (1)	To a high degree (2)	Somewhat (3)	To a low degree (4)	To a very low degree (5)
Is your work emotionally exhausting? (1)	○	○	○	○	○
Do you feel burnt out because of your work? (2)	○	○	○	○	○
Does your work frustrate you? (3)	○	○	○	○	○

Q71 Please rate your work-related burnout.

	Always (1)	Often (2)	Sometimes (3)	Seldom (4)	Never/almost never (5)
Do you feel worn out at the end of the working day? (1)	○	○	○	○	○
Are you exhausted in the morning at the thought of another day at work? (2)	○	○	○	○	○
Do you feel that every working hour is tiring for you? (3)	○	○	○	○	○
Do you have enough energy for family and friends during leisure time? (4)	○	○	○	○	○

End of Block: Personal Health and Well-Being

Start of Block: Work-Life Balance Functioning

Q31 Considering your work <u>and</u> personal life: **In the past**, when you encounter difficulties, what is the source that you ever received **comfort** and **caring**? (Select all that apply)

- ☐ No source (1)
- ☐ Spouse/Partner(s) (2)
- ☐ Other family members (3)
- ☐ Friends (4)
- ☐ Relatives (5)
- ☐ Colleagues (6)
- ☐ Companies (7)
- ☐ Official or semi-official organizations, such as, parties, leagues and trade union (8)
- ☐ Unofficial organizations, such as, religion, social group and etc. (9)
- ☐ Others (please specify) (10) ____

Q32 Considering your work <u>and</u> personal life: **In the past**, when you encounter difficulties, what is the source that you ever received either **economic support** or **practical problem-solving** help? (Select all that apply)

▢ No source (1)

▢ Spouse/Partner(s) (2)

▢ Other family members (3)

▢ Friends (4)

▢ Relatives (5)

▢ Colleagues (6)

▢ Companies (7)

▢ Official or semi-official organizations, such as, parties, leagues and trade union (8)

▢ Unofficial organizations, such as, religion, social group and etc. (9)

▢ Others (please specify) (10)

Display This Question:

If If How many people currently reside in your household including yourself? Children (less than 18 years old) Is Not Equal to 0

Q33 For households with children, what type of child care arrangement does your family use currently? (Select all that apply)

- ▢ Cared for in our home by parents/family/sitter (1)
- ▢ Informal arrangement with neighbors/family (2)
- ▢ Child care center/ preschool (3)
- ▢ Home-based care (4)
- ▢ School (K-12) (5)
- ▢ Homeschooled or virtual school (K-12) (6)
- ▢ Other (please specify) (7) ____
- ▢ Children are of age that do not require childcare (8)
- ▢ Prefer not to say (9)

Display This Question:

If If How many people currently reside in your household including yourself? Children (less than 18 years old) Is Not Equal to 0

Q34 For households with children, who is the primary party in your household responsible for childcare?

- ◯ Self (1)
- ◯ Spouse/partner (2)
- ◯ Equal division between self and spouse/partner (3)
- ◯ Other (please specify): (4) ____

Q35 Are you or anyone in your household caring for a sick family member or providing elder care?

- ○ Yes (1)
- ○ No (2)
- ○ Unsure (3)
- ○ Prefer not to say (4)

Q36 Do you or anyone in your household have a medical condition that increases risk for sever illness from COVID-19 [i.e., cancer, chronic kidney disease, COPD, immunocompromised state (weakened immune system), obesity (body mass index [BMI] of 30 or higher), serious heart conditions, sickle cell disease, type 2 diabetes mellitus]?

- ○ Yes (1)
- ○ No (2)
- ○ Unsure (3)
- ○ Prefer not to say (4)

Q37 What do you feel is the level of your financial stress today?

- ○ Overwhelming stress (1)
- ○ Above average stress (2)
- ○ Average stress (3)
- ○ Some stress (4)
- ○ No stress at all (5)

Page Break

Q38 How often do you?

	Frequently (1)	Occasionally (2)	Rarely (3)	Never (4)	It never occurred to me (5)
Eat regularly (e.g. breakfast, lunch, and dinner) (1)	○	○	○	○	○
Eat healthy foods (2)	○	○	○	○	○
Exercise consistently (3)	○	○	○	○	○
Get regular medical care for prevention (4)	○	○	○	○	○
Get medical care when necessary (5)	○	○	○	○	○
Take time off when necessary (6)	○	○	○	○	○
Take time off when sick (7)	○	○	○	○	○
Feel you can take time off from work (8)	○	○	○	○	○
Get enough sleep (9)	○	○	○	○	○

Q39 In a week, how many hours did you work **pre-pandemic** vs. **the past week**?

________ **Pre-Pandemic** number of hours (1)

________ In the **past week** number of hours (2)

Q40 How many days a week did/do you typically work **pre-pandemic** and **now**?
________ Days per week **pre-pandemic** (1)
________ Days per week **now** (2)

Page Break

Q41 Have you or anyone within your agency received any personal threats regarding COVID-19?

- ○ Yes (1)
- ○ No (2)
- ○ Unsure (3)
- ○ Prefer not to say (4)

Skip To: Q43 If Have you or anyone within your agency received any personal threats regarding COVID-19? = No

Skip To: Q43 If Have you or anyone within your agency received any personal threats regarding COVID-19? = Unsure

Skip To: Q43 If Have you or anyone within your agency received any personal threats regarding COVID-19? = Prefer not to say

---

Q42 If yes, from whom were these threats received? Please be specific as you are comfortable (select all that apply).

- ▢ A member of the public (1)
- ▢ Someone from within your agency or organization (2)
- ▢ Someone from outside your agency but in a leadership (e.g., government or administrative role) (3)
- ▢ Other (please describe): (4) ____

Q63 If yes, please describe the threats.

____

____

____

____

____

Q43 Have you or anyone within your agency received any political pressure or political threats?

- Yes (1)
- No (2)
- Unsure (3)
- Prefer not to say (4)

Skip To: Q45 If Have you or anyone within your agency received any political pressure or political threats? = No

Skip To: Q45 If Have you or anyone within your agency received any political pressure or political threats? = Unsure

Skip To: Q45 If Have you or anyone within your agency received any political pressure or political threats? = Prefer not to say

Q75 If yes, from whom were these political pressures or threats received? Please be specific as you are comfortable (select all that apply).

- A member of the public (1)
- Someone from within your agency or organization (2)
- Someone from outside your agency but in a leadership position (e.g., government or administrative role) (3)
- Other (please describe): (4) ____

Q44 If yes, please describe the threats.

____

Page Break

Q45 During the COVID-19 response, which of the following has your workplace adopted as safety measures? (Select all that apply)

- ☐ Required masks (1)
- ☐ Required face shields/protection (2)
- ☐ Social distancing indoors (3)
- ☐ Social distancing outdoors (4)
- ☐ Adapted food safety practices (5)
- ☐ Telework options (working remotely) (6)
- ☐ Flexible work schedules / rotations (7)
- ☐ Additional cleaning procedures (8)
- ☐ Other, (please describe) (9)

____

Q46 How have these safety measures been implemented?

- ○ Continuously since the start of the pandemic (1)
- ○ Sporadically since the start of the pandemic (2)
- ○ Available once at the beginning, and not since (3)
- ○ Other (please specify): (4) ____

Q47 During the COVID-19 response, which of the following has your workplace adopted as supportive measures for employees? (Select all that apply)

- ▢ Free or low-cost mental health services (1)
- ▢ Financial support for mental health services (2)
- ▢ Free or low-cost childcare or eldercare services (3)
- ▢ Financial support for childcare or eldercare services (4)
- ▢ Stress-reduction tips or tools (5)
- ▢ Free meals or food options for in-person staff (6)
- ▢ Other (please specify): (7) ____

Q48 How have these support measures been implemented?

- ○ Continuously since the start of the pandemic (1)
- ○ Sporadically since the start of the pandemic (2)
- ○ Available once at the beginning, and not since (3)
- ○ Other (please specify): (4) ____

Q76 Have you personally used any of the supportive measures available to you?

- ○ Yes (1)
- ○ No (2)
- ○ Prefer not to say (3)

Skip To: Q68 If Have you personally used any of the supportive measures available to you? = No

Skip To: Q68 If Have you personally used any of the supportive measures available to you? = Prefer not to say

Q77 If yes, how helpful have you found these supportive measures? (1: not helpful at all to 10: exceptionally helpful)

________ Supportive measures (1)

Q68 What one (1) supportive measure could your workplace implement to help you most right now?

____

Q49
Are there any best practices observed in the workplace that you would like to highlight (e.g., an online chat communication platform like Skype/Google for communication)? Please be as specific as possible.

____

____

____

____

____

Q50 During the response to COVID-19, what routine duties or services have/are not able to be done (i.e., what pre-pandemic public health services are not getting done now, like foodborne investigations)? Please be as specific as you feel comfortable.

____

____

____

____

Q51 Do you have any specific suggestions or feedback about this assessment regarding the questions that were asked or any that were absent? Please be as specific as possible.

____

____

____

____

____

Q52 Would you be willing to participate in a phone or virtual interview as a key informant?

○ Yes (1)

○ No (2)

Q53 Thank you for your participation in our survey. The information provided will be valuable to us.

End of Block: Work-Life Balance Functioning
